# In Vitro and In Silico Cytotoxic Activity of Isocordoin from *Adesmia balsamica* Against Cancer Cells

**DOI:** 10.3390/ijms26052238

**Published:** 2025-03-02

**Authors:** Valentina Silva, Evelyn Muñoz, Catalina Ferreira, Alessandra Russo, Joan Villena, Iván Montenegro, Daniela Birchmeier, Alejandro Madrid

**Affiliations:** 1 Laboratorio de Productos Naturales y Síntesis Orgánica (LPNSO), Facultad de Ciencias Naturales y Exactas, Universidad de Playa Ancha, Leopoldo Carvallo 270, Playa Ancha, Valparaíso 2340000, Chile; silvapedrerosv@gmail.com (V.S.); evdmunoz@gmail.com (E.M.); cata.ferreira.funes@gmail.com (C.F.); 2 Department of Drug and Health Sciences, University of Catania, V.le A. Doria 6, 95125 Catania, Italy; alrusso@unict.it; 3 Center of Interdisciplinary Biomedical and Engineering Research for Health (MEDING), Escuela de Medicina, Facultad de Medicina, Universidad de Valparaíso, Angamos 655, Reñaca, Viña del Mar 2520000, Chile; juan.villena@uv.cl; 4 Center of Interdisciplinary Biomedical and Engineering Research for Health (MEDING), Escuela de Obstetricia y Puericultura, Facultad de Medicina, Universidad de Valparaíso, Angamos 655, Reñaca, Viña del Mar 2520000, Chile; ivan.montenegro@uv.cl; 5 Millennium Nucleus Bioproducts, Genomics and Environmental Microbiology (BioGEM), Avenida España 1680, Valparaíso 2390123, Chile; 6 Unidad de Citogenética, Hospital Gustavo Fricke, Avenida Álvarez 1532, Viña del Mar 2520000, Chile; d.birchmeier.s@gmail.com

**Keywords:** *Adesmia balsamica*, isocordoin, cytotoxic, apoptosis, caspase, molecular docking

## Abstract

This study investigates the anticancer potential of isocordoin, a prenylated chalcone found in *Adesmia balsamica*. In vitro assays on colorectal (HT-29), breast (MCF-7) and prostate (PC-3) cancer cell lines, together with a non-cancerous colon cell line (CoN CCD841), revealed that isocordoin is cytotoxic, with PC-3 and MCF-7 cells showing the highest sensitivity. The selectivity index was higher for PC-3 (5.2) than for MCF-7 (3.7) and HT-29 (2.9). Isocordoin induced morphological changes suggestive of apoptosis in tumor cells. Mechanistic studies on HT-29 and MCF-7 lines indicated that isocordoin might possess antioxidant properties while promoting the loss of mitochondrial membrane potential and caspases activation. Molecular docking showed a favorable interaction of isocordoin with caspase-3, which could explain its apoptotic effects. In silico predictions suggest that isocordoin has drug-like properties, including good absorption and permeability to the blood-brain barrier. The presence of the prenyl chain in isocordoin appears crucial for cytotoxic activity, supported by its higher lipophilicity and better interaction with caspase-3 compared to non-prenylated 2′,4′-dihydroxychalcone. Overall, isocordoin demonstrates promising anticancer activity, warranting further investigation as a potential therapeutic agent.

## 1. Introduction

**Figure 1:**
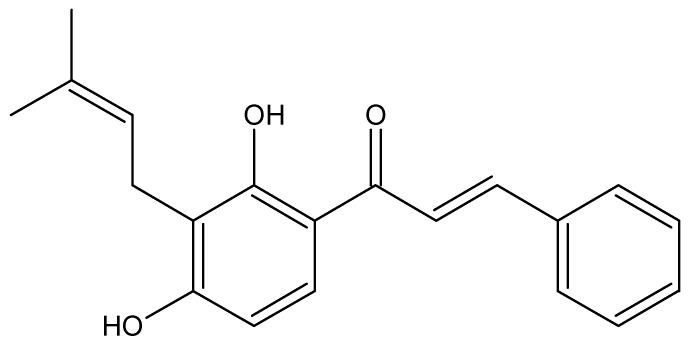
Isocordoin structure.

Cancer is a non-communicable disease that causes a number of social, public health, and economic problems in the 21st century [1]. Considering the widespread impact of this disease, strategies for its prevention and treatment are constantly being sought. ROS-mediated mechanisms have been a target for therapies in cancer treatment as there are pathways by which ROS can lead to cancer cells death, one example being apoptosis or programmed death. The same is true for caspases, as one of the signaling pathways in the process of apoptosis is their activation [2]. Advances in the understanding of the biological, genetic, and molecular factors of cancer have allowed for the development of more specific forms of therapy, reducing side effects [3]. The search for new therapeutic approaches also aims to improve the selectivity of anticancer agents, avoiding damage to non-target tissues, with greater or equal effectiveness. For this purpose, attention has been placed on natural compounds, which can be used in cancer therapy and even as chemo-preventive compounds [4]. Due to their reported antioxidant properties [5] and ROS-related therapeutic properties [6,7], a variety of natural products are of interest for use alone or in combination with modern cancer therapies. This biological potential has been the driving force behind the search for bioactive metabolites in natural products. Among these metabolites is the chalcone family, which are the precursors of flavones in flavonoid biosynthesis. The skeleton of chalcones triggered interest in researchers due to their use as a template for medicinal chemistry, allowing for a wide variety of substitutions and syntheses for drug development [8]. An interesting subgroup of prenylated chalcones of natural origin stand out for their lipophilicity, some of them are isobavachalcone, xanthoangelol and isoxanthoangelol, which are cytotoxic [9,10,11]. This type of chalcone can be found in plants belonging to the Leguminosae family, especially in the genus *Adesmia* (Fabaceae), among them isocordoin (Figure 1).

This molecule has significant bioactivity and has been reported to have antioomycete, antiprotozoal, anti-feedant, anti-inflammatory, anti-hypersensitive, antioxidant and vasorelaxant effects [12,13,14,15,16,17]. Isocordoin also has cytotoxic activity in cell lines of oral-laryngeal and prostate cancers [18]. In addition, isocordoin analogues have been shown to promote apoptosis in human melanoma cells through Hsp70 [19]. According to what has been described, it can be said that isocordoin has a wide therapeutic utility in which the anticancer activity stands out.

Based on this background, the objective of this research was to evaluate the cytotoxic activity of isocordoin in three different cancer cell lines: colorectal adenocarcinoma (HT-29), mammary ductal carcinoma (MCF-7), grade IV prostate adenocarcinoma (PC-3) selected because of their clinical relevance, and in a non-cancerous colon epithelial cell line (CoN CCD841). After establishing its cytotoxic effect, the mechanism of action of this compound in two selected cell lines (MCF-7 and HT-29) was deciphered by evaluating changes in ROS production, mitochondrial membrane permeability, and caspases activation. Finally, the molecular coupling of isocordoin in the catalytic region of caspase-3 and an in silico prediction of the physicochemical, pharmacokinetic, and profile properties was performed.

## 2. Results and Discussion

The cytotoxic activity of isocordoin was evaluated against human colorectal adenocarcinoma (HT-29), breast adenocarcinoma (MCF-7), prostate adenocarcinoma (PC-3), and normal colon epithelial (CoN CCD841) cell lines in vitro. The results are summarized in Table 1, with daunorubicin and 5-fluorouracil (5-FU) used as positive controls in the experiments. Additionally, the selectivity index for cancer cell lines was calculated from the ratio of their respective IC_50_ values and that of CoN CCD841 normal cell (Table 1).

**Figure 2:**
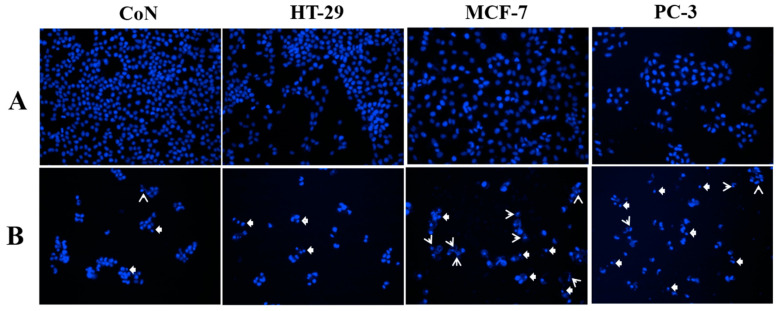
Effect of isocordoin on cellular morphology, chromatin condensation and fragmentation, of CoN CCD841, HT-29, MCF-7, and PC-3 cell lines: (**A**) control cells exposed to 1% ethanol; (**B**) cells treated with isocordoin (50 μM) for 48 h. Arrow heads and arrows indicate not condensed nuclei and condensed and/or fragmented nuclei, respectively.

Based on the IC_50_ values presented in Table 1, isocordoin demonstrated the ability to decrease cell viability across all cancer cell lines tested. Notably, this chalcone displayed a significant cytotoxic effect against PC-3 and MCF-7 cells, with IC_50_ values of 15.2 µM (SI = 5.2) and 21.1 µM (SI = 3.4), respectively. These results are quite comparable to the effects of 5-FU. While isocordoin exhibited a moderate cytotoxic effect on HT-29 cells, with an IC_50_ of 27.2 µM (SI = 2.9), compared to the more potent 5-FU (2.9 µM), it was comparable to daunorubicin (15.1 µM). It is important to note that daunorubicin exhibited high toxicity against healthy cell lines and lacked selectivity towards malignant cells. Furthermore, a qualitative observation of morphological changes in cell lines treated with 50 µM isocordoin revealed that tumor cell lines displayed altered morphology (Figure 2), exhibiting a reduction in size and loss of their fusiform shape, adopting a more rounded appearance.

Figure 2 illustrates that nuclear morphology in control cell lines was preserved in comparison with isocordoin-treated cells, which showed condensation (arrows) and/or fragmentation (arrow head) of chromatin in all cell lines used; this could be associated with cell death processes by apoptosis. To find out which is the mechanism of action, the cell lines HT-29 and MCF-7 were selected for further testing. PC-3 was not included in the mechanism of action elucidation, since it has been previously determined [20] and both positive controls used prove to be selective, unlike the other tested cell lines. To elucidate the role of reactive oxygen species (ROS) in isocordoin-induced cell death, intracellular levels of ROS were measured. ROS production and protein release from the mitochondrial intermembrane space have been linked to the activation of multiple cell death mechanisms. According to Table 2, which displays the production of ROS, the mechanism by which isocordoin shows an increase in fluorescence involves the ROS-dependent conversion of non-fluorescent 2′,7′-dichlorofluorescein diacetate (DCFH-DA) to the fluorescent product 2′,7′-dichlorofluorescein (DCF).

Isocordoin showed a slight effect on fluorescence in the MCF-7 cell line, with a minimal increase at 25 µM and no difference with the control at 50 µM. In contrast, in the HT-29 cell line, a slight increase in fluorescence was observed at 25 µM. However, the production of ROS in HT-29 was significantly higher at both isocordoin concentrations, reaching 45.5% at 50 µM. These results, although modest, suggest that isocordoin may have antioxidant properties, which is relevant. Similar to ubiquinone analogues, it could act as a cytoprotective agent at the mitochondrial level [21], or alternatively, it could exert an effect similar to that of the compound 9,10-dihydroxy-4,4-dimethyl-5,8-dihydroanthracen-1(4H)-one, which decreases ROS generation and shows intracellular antioxidant properties that inhibit cell viability by non-oxidative mechanisms [22].

This is particularly relevant because the mitochondrial membrane potential, stemming from the electrical potential created by the electrochemical gradient across the inner mitochondrial membrane, is sensitive to changes in permeability, which can trigger apoptosis [23]. In accordance with histograms obtained by flow cytometry using Rhodamine 123 (see the Supplementary Material), isocordoin achieves a decrease in this fluorescence, which indicates a loss of the mitochondrial membrane potential in the treated HT-29 and MCF-7 cell lines (Table 3).

The analysis of mitochondrial function revealed that isocordoin treatments induce a significant impairment, expressed in a marked loss of membrane potential. This alteration in mitochondrial membrane potential is found to be associated with cell viability results, showing a correlation with reduced viability and morphological alterations of the cells. Considering the structural properties of isocordoin, it may have a protonophore effect, meaning that it is capable of translocating protons across lipid bilayers, such as the mitochondrial membrane. As mentioned above, the presence of the α, β-unsaturated system and the substitution with the hydroxyl group in the ring may confer this property and allow it to uncouple the process of proton gradient (Δp) generation from ATP synthesis [24,25].

Suppression of the apoptotic machinery is one of the distinctive features of cancer, in the cell death process, the activation of caspases, plays a key role [21]. To determine if isocordoin acts on the activation of caspases, a marker (CaspACE™) who binds to active caspases was used. For this case, the fluorescence is proportional to the activation of caspases and, therefore, to the induction of apoptosis. As shown in Table 4, there was an increase in caspase activation in the case of the HT-29 line.

### 2.1. In Silico Approach

#### 2.1.1. Molecular Docking

In terms of bioactivity of chalcones like isocordoin, it has been concluded that the presence of at least one phenolic hydroxyl group, especially at position 4′ of the B-ring and a certain degree of lipophilicity with the presence of isoprenyl side chain in the substitution pattern are important for bioactivity [26]. To support the evidence that the prenyl chain would confer greater lipophilicity and thus greater activity, molecular docking to caspase-3 and in silico approach to the pharmacokinetics and pharmacodynamics of isocordoin were performed and compared with a chalcone that differs from isocordoin only in the presence of the prenyl chain 2′,4′-dihydroxychalcone.

Before performing the docking of the compounds listed in Table 5, the AutoDock software was tested by re-docking the native ligand, 2-hydroxy-5-(2-mercaptoethylsulfonamide) benzoic acid, into the binding pocket of caspase-3. The RMSD obtained as a result of applying this method was equal to 1.62 Å between the docked pose and the pose corresponding to the 3D crystal structure. This degree of variation was determined to be acceptable for the validation of the docking procedure.

**Figure 3:**
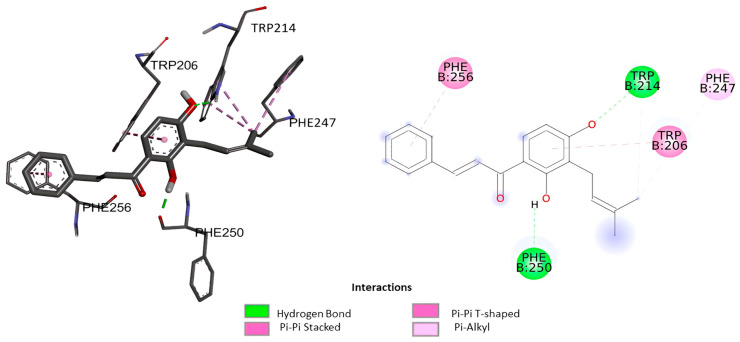
Three-dimensional and 2D interaction of isocordoin with the binding pocket of caspase-3.

**Figure 4:**
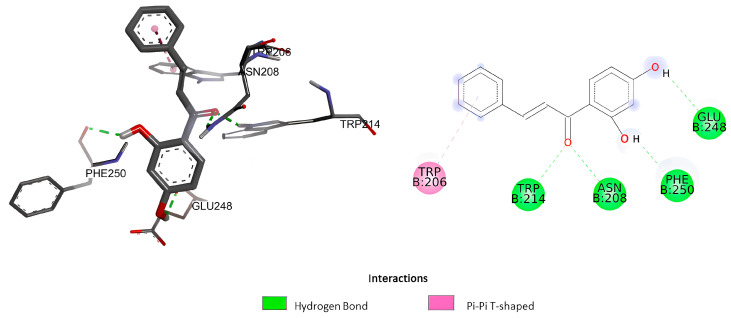
Three-dimensional and 2D interaction of 2′,4′-dihydroxychalcone with the binding pocket of casapase-3.

**Figure 5:**
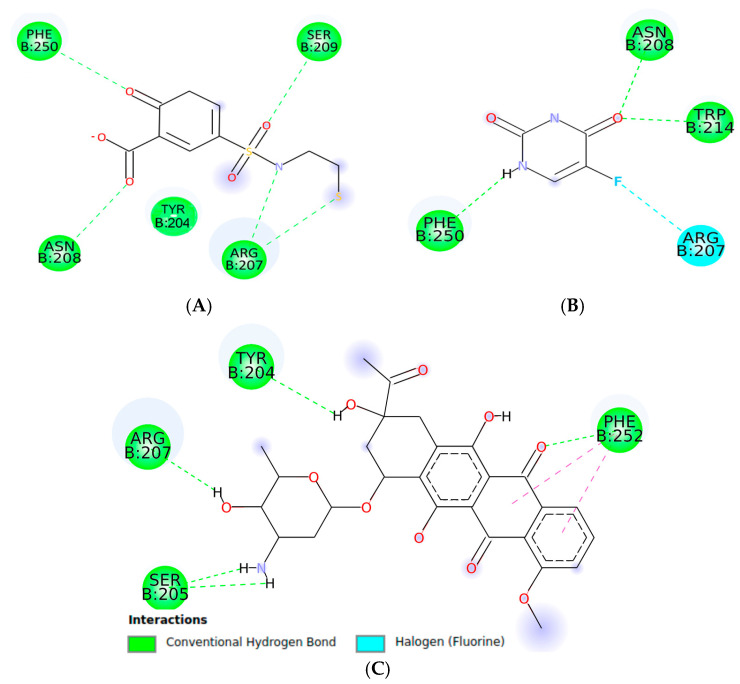
Two-dimensional interaction of 2-Hydroxy-5-(2-mercaptoethylsulfamoyl)-benzoic acid (**A**), 5-FU (**B**), and daunorubicin (**C**) with the binding pocket of casapase-3.

The docking analysis of isocordoin, 2′,4′-dihydroxychalcone, as well as the compounds used as controls, are shown in Figure 3, Figure 4 and Figure 5.

Table 5 presents the values obtained from the molecular docking of the analyzed compounds on the caspase-3 receptor. To conduct the molecular docking study, the rigid crystal structure of caspase-3 (PDB ID: 1NME) was employed. This structure was selected for its high structural quality, determined through X-ray crystallography, which provides high-resolution data and an accurate representation of the receptor’s conformation. Furthermore, as a crystal structure, the residues in the binding pocket are well-defined, facilitating the modeling of interactions between the ligand and the receptor.

The value of the binding energy of isocordoin was −6.13 kcal/mol, while that of the 2′,4′-dihydroxychalcone was −5.58 Kcal/mol. These results indicate that the docking of isocordoin is more stable and effective in the caspase-3 binding pocket than the 2′,4′-dihydroxychalcone, while the binding energies of the control compounds were −7.09 Kcal/mol for daunorubicin and −3.68 for 5-FU. The inhibition constant (Ki) obtained through AutoDock 4.2 is an estimate of the binding affinity between a ligand and a target protein under ideal conditions. Low values of Ki indicate a high affinity between the ligand and the enzyme. According to the results obtained, daunorubicin exhibits the highest affinity, with a Ki value of 6.33 µM, followed by isocordoin, which has a Ki of 32.01 µM. In contrast, 5-FU is the compound with the lowest affinity, as its Ki is the highest among those analyzed. The compounds NLBA and 2,4’-dihydroxychalcone fall within an intermediate range and may require further studies to evaluate their potential.

As can be seen in Figure 3, the interactions generated between isocordoin and caspase-3 correspond to two hydrogen bonds between the hydroxyl groups of the aromatic ring A and residues Trp214 and Phe250 of caspase-3. Some lower strength pi-type interactions are also observed with residues Phe256, Trp206, and Phe247. The 2′,4′-dihydroxychalcone formed four hydrogen bonds, as shown in Figure 4, two of them between the hydroxyl groups of the A ring and the residues of Phe250 and Glu248, while the carbonyl group of the molecule formed two simultaneous hydrogen bonds with the residues Asn208 and Trp214. These interactions are important because they reveal that isocordoin could bind to the caspase-3 and experimental studies expose that it also acts as an activator. The caspase family consists of more than twelve proteases characterized by the presence of cysteine residues in their active site. These proteins are highly conserved throughout the evolution and share common structural sequences [27]. Caspases are synthesized as inactive precursors that are converted to the active form by proteolytic cleavage. Once active, caspases produce hydrolysis from aspartic acid residues in the substrate protein. Thus, the initial activation of one caspase sets off a chain reaction that leads to the activation of other caspases and cell death. The regulation of caspase activation is therefore essential for cell survival [28]. Additionally, caspase-3 plays a key executioner role, and its inhibition can drastically prevent apoptosis in vitro and in vivo [29].

#### 2.1.2. Prediction of Physicochemical Properties, Pharmacokinetics, and Drug-likeness Profile

In clinical trials, the choice of a new drug is considered very complicated due to the inadequate properties of ADME (absorption, distribution, metabolism, and excretion), in addition to the costs required to develop a new drug. The evaluation of the pharmacokinetic and pharmacodynamic properties of a chemical compound is a critical step in the development process of a new drug. We evaluated these parameters of isocordoin and 2,4’-dihydroxychalcone using in silico methods (Table 6 and Table 7).

Table 6 and Table 7 reveal that both compounds comply with Lipinski’s rules, since both have a molecular weight of less than 500 g/mol, a lipophilicity (Log P) of less than 5, less than 10 rotatable bonds, and a number of hydrogen bond donors less than 5 [30]. In theory, they would have strong oral absorption, high gastrointestinal absorption, and the ability to cross the blood-brain barrier. Log S indicates moderate water solubility for isocordoin and high solubility for 2′,4′-dihydroxychalcone. Finally, the model score for bioavailability is positive for both cases (0.55).

Literature on anticancer chalcones as isocordoin highlights the substitution with hydroxyls in the ring (A and B) as favoring their anticancer capacity. Also, the α, β unsaturated carbonyl functional group facilitates their association with thiol groups such as cysteine residue, an interaction that is key to the bioactivity of these molecules [31]. Also, as demonstrated in the in silico assays, the prenyl chain confers higher lipophilicity for isocordoin and thus higher activity versus the non-prenylated chalcone 2′,4′-dihydroxychalcone. This is consistent with other studies that have shown that 2′,4′-dihydroxychalcone is inactive in all three cell lines used in this study with IC_50_ values close to 100 μM [32], showing a Log P of 2.75 versus 4.24 for isocordoin.

## 3. Materials and Methods

### 3.1. Plant Material

Aerial parts of *Adesmia balsamica* Bertero ex Colla were collected at Viña del Mar (Valparaíso Region, Chile) in Summer 2024. A voucher specimen is kept in the Herbarium of Natural Products Laboratory of Universidad de Playa Ancha, Valparaíso, Chile (ABVM-2024).

### 3.2. Extraction and Isolation of Isocordoin

The fresh material (1.0 Kg) of *A. balsamica* was immersed in cold dichloromethane for 40 s at room temperature. The solution was concentrated at reduced pressure in a rotary evaporator to yield a resinous exudate (97 g).

The resinous exudate of *A. balsamica* (45 g) was fractionated by column chromatography (C.C., 12 cm diameter, 50 cm length, ground-glass mouth 29/32, with Teflon key) on silica gel (Merck 200–300 mesh) eluting with mixtures of petroleum ether and ethyl acetate of increasing polarity (*v*/*v* 100:0–70:30). The progress of separation of isocordoin was monitored and analyzed by thin-layer chromatography (TLC). TLC spots were detected by heating after spraying with 25% H_2_SO_4_ in H_2_O. After C.C., pure isocordoin was isolated as a yellow solid (395 mg, 0.88%). The structure was confirmed by comparing its spectroscopic data with those reported in the literature [16].

### 3.3. Cell Lines and Culture Conditions

In this study, we used three different tumor cell lines: human breast adenocarcinoma (MCF-7), human prostate adenocarcinoma (PC-3), and human colorectal adenocarcinoma (HT-29). A non-tumoral human cell line of colon epithelial cells (CCD 841 CoN) was included to evaluate selectivity. All tested cell lines were obtained from the American Type Culture Collection (Rockville, MD, USA). The different cell lines were maintained as monolayers in a culture medium (HAM-F10 + DMEM, 1:1) supplemented with 10% fetal bovine serum, as well as antibiotics (0.01 mg/mL streptomycin and 0.005 mg/mL penicillin). The cells were incubated at 37 °C in a humidified 5% CO_2_ atmosphere.

### 3.4. Cytotoxicity Assay

To determine the effect on cell viability of the samples, the sulforhodamine B (SRB) assay was used to measure the number of viable cells after each treatment [32]. Cells were seeded in 96-well plates at a density of 3 × 10^3^ cells/well in 100 µL of culture medium and treated in triplicate at concentrations of 5, 10, 25, 50, and 100 µM. After each incubation, the cells were fixed with trichloroacetic acid, then washed by immersion in distilled water and stained with 50 µL/well of SRB, as well as 0.1% (*w*/*v*) in 1% (*v*/*v*) acetic acid at room temperature for 30 min. The dye is solubilized with 150 µL/well of 10 mM Tris Base, and the absorbance is then measured at a wavelength of 540 nm in a microplate reader.

### 3.5. Selectivity Index

The selectivity index (SI) was determined by the ratio between the IC_50_ value of the cytotoxicity obtained for CCD 841 CoN cells and the value found for a selected cancer cell line, as shown in Equation (1):

SI = IC_50_ (CoN cells)/IC_50_ (cancer cell line) (1)

where a SI > 3 was considered to belong to a selective sample [33].

### 3.6. Cellular and Nuclear Morphology

To qualitatively examine the morphological changes, cell lines were treated with 50 µM of isocordoin for 48 h. Phase contrast was used to observe the phenomena of cell morphology modification. Cells were labeled with Hoechst dye for 30 min at 37 °C and visualized with DAPI filter [32]. Photographs were captured at 20x.

### 3.7. Reactive Oxygen Species (ROS) Generation

Cells were seeded in 24-well plates at a density of 16∙10^4^ cells/well in 500 µL of culture medium. The determination of ROS was performed following the methodology of Villena et al. (2021) using a fluorescent probe 2′,7′-dichlorofluorescein diacetate (DCFH-DA) [32]. The cells were treated with isocordoin at a concentration of 25 µM and 50 µM. Subsequently, cells were labeled with DCFH-DA for one hour at 37 °C in an incubator. Untreated cells were used as unlabeled control. In addition, solvent-treated and DCFH-DA-labeled cells were used as control. Fluorescence was measured by flow cytometry using the FL1 filter. The results obtained after the flow cytometer analysis were plotted in histograms and analyzed to evaluate the production of reactive oxygen species, using the Cyflogic 1.2.1 software.

### 3.8. Mitochondrial Membrane Permeability

The methodology for assessing the mitochondrial membrane potential (ΔΨm) change was adapted from the protocols described previously [32]. Cells were seeded in 24-well plates at a density of 5 × 10^4^ cells/well in 500 µL of culture medium. After 24 h of incubation, the cells were treated with the compound at 25 µM and 50 µM for 48 h. After treatment, 5 µL of Rhodamine 123 was added to each well and incubated for 1 h at 37 °C. Rhodamine 123-associated fluorescence was analyzed by flow cytometry, using the FL1 filter to evaluate the percentage of cells with decreased mitochondrial membrane potential, using the Cyflogic software 1.2.1.

### 3.9. Caspase Activation

In caspase activation, assessment cells were treated with the FITC-VAD-FMK marker (CaspACE™) and seeded in 24-well plates at a density of 8 × 10^4^ cells/well in 500 µL of culture medium. After 24 h of incubation, the cells were exposed to isocordoin at a concentration of 25 µM and 50 µM for 48 h. After this time, labeling was performed for 1 h at 37 °C in the dark with the CaspACE marker. Untreated cells were used as unlabeled control. In addition, solvent-treated and CaspACE-labeled cells were used as control. The histograms obtained after the flow cytometer analysis using the FL1 filter were analyzed to evaluate the percentage of cells with caspase activation, using the Cyflogic 1.1 software [34].

### 3.10. In Silico Assays

#### 3.10.1. Materials

A computer equipped with an Intel^®^ Core™ i7-processor, 7200U CPU @2.50 GHz (4 CPUs. 2.7 GHz), 16 GB of RAM, Ubuntu 18.04 64-bit, and Windows 10 Pro 64-bit operating system was used for molecular docking.

#### 3.10.2. Construction of Ligands

The three-dimensional models of the ligands were built using the Avogrado 1.2.0.n software. The geometry of the ligands was optimized and their energy was minimized using the MMFF94 force field.

#### 3.10.3. Molecular Docking

Molecular docking of isocordoin in the catalytic region of caspase-3 was evaluated, and the 3D crystallographic structure of caspase-3 (PDB ID: 1NME) with 2-hydroxy-5-(2-mercapto-ethylsulfamoyl)-benzoic acid as a natural ligand (NLBA) were obtained from the Protein Data Bank (RSCB) website [35]. The crystal structure of the enzyme was edited in Discovery Studio Visualizer software (v17.2.0.16349 2016), in which ligands and water molecules associated with the PDB file were removed. The docking method was carried out using the AutoDock 4.2 software, using the Lamarckian Genetic Algorithm [36] and assuming rigid ligands in the macromolecule and full flexibility for the ligand. Kollman to the enzyme, and Gasteiger charges were added to the ligand in addition to the polar hydrogens. The grid coordinates were 40.881 Å (X), 95.736 Å (Y) and 24.611 Å (Z), while the grid box sizes were 45 grid points (X), 40 grid points (Y) and 40 grid points (Z) [37]. The search parameters were 50 runs, with a maximum number of 25,000,000 evaluations for each ligand. The RMSD threshold was set for multiple clusters at <0.5 Å. The results were ordered according to the binding energy and possible conformations. The lowest binding energy and most likely conformation were chosen for analysis. The Ki value was calculated from the binding free energy (ΔG) using the following Equation (2):

Ki = ΔG/*e*^RT^ (2)

where ΔG is the binding free energy (kcal/mol) obtained from AutoDock, R is the universal gas constant (1.987 cal/(mol·K)), and T is the temperature in Kelvin (default: 298.15 K).

The Discovery Studio Visualizer software (version 21.1.0.20298) was used to acquire a two-dimensional, 3D image of the most stable conformation chosen.

#### 3.10.4. Prediction of Physicochemical Properties, Pharmacokinetics, and Drug-likeness Profile

The physicochemical and lipophilicity of the target compounds were collected using the Swiss ADME software version 2025 (http://www.swissadme.ch/index.php, accessed on 5 January 2025). The percentage of absorption was calculated through Equation (3):

%ABS = 109 − 0.345 tPSA,(3)

where TPSA is the topological polar surface area and %ABS denotes the percentage of absorption.

## 4. Conclusions

In summary, the structure of isocordoin, particularly the iso-prenyl chain, is essential for its bioactivity and solubility. The in silico study confirmed the importance of this chain for the interaction with caspase-3. The anticancer activity of isocordoin, by inducing apoptosis, makes it a promising candidate for the development of future cancer therapies.

## Figures and Tables

**Table 1:** IC_50_ values (μM) and selectivity index (SI) of cancer cell lines treated with isocordoin.

Sample	Cell lines IC_50_ (µM) ^a^ (SI ^b^)			
	HT-29	MCF-7	PC-3	CoN CCD841
**Isocordoin**	27.2 ± 0.3 * (2.9)	21.1 ± 0.2 * (3.7)	15.2 ± 0.6 * (5.2)	78.7 ± 0.1
**Daunorubicin**	15.1 ± 0.5 (<1)	0.33 ± 0.2 (42)	0.41 ± 0.4 (33.9)	13.9 ± 0.3
**5-FU**	2.9 ± 0.7 (19.3)	22.3 ± 0.2 (2.5)	16.4 ± 0.6 (3.4)	56.1 ± 0.5

^a^ Values expressed as the mean values ± standard deviation of three independent experiments, each preformed in triplicate; ^b^ Selectivity index. * *p* < 0.05 vs. control (ethanol-treated cells).

**Table 2:** Percentage of ROS-positive cells after treatment with isocordoin.

Sample	Concentration (µM)	HT-29	MCF-7
Isocordoin	25	27.8 ± 1.9 *	5.0 ± 1.1
	50	45.5 ± 3.7 *	8.3 ± 1.2
Control ^a^	---	15.7 ± 0.9	6.5 ± 0.9

^a^ Control: ethanol solvent-treated cells. * *p* < 0.05 vs. control cells.

**Table 3:** Changes induced by the treatment with isocordoin, in mitochondrial membrane permeability of HT-29 and MCF-7 cells. Permeability changes are measured as the percentage of Rhodamine 123 stained cells found in cell lines treated with isocordoin (25 and 50 µM) against control cells (0.1% DMSO).

Sample	Concentration (µM)	HT-29	MCF-7
Isocordoin	25	40.8 ± 5.1 *	37.7 ± 2.2 *
	50	37.0 ±2.9 *	54.9 ± 6.1 *
Control	---	7.6 ± 0.9	13.2 ± 1.6

Percentage of cells stained with Rhodamine 123 after the treatment with isocordoin. * *p* < 0.05 vs. control cells (ethanol-treated cells).

**Table 4:** Caspase-3 activity percentage of HT-29 and MCF-7 cells after the treatment with isocordoin.

Sample	Concentration (µM)	HT-29	MCF-7
Isocordoin	25	22.8 ± 1.4 *	8.3 ± 1.0
	50	27.7 ± 1.6 *	25.6 ± 2.6 *
Control ^a^	---	13.6 ± 1.1	8.8 ± 0.9

Percentage of cells with caspases activated after the treatment with isocordoin. * *p* < 0.05 vs. ^a^control cells (ethanol-treated cells).

**Table 5:** Docking score and estimated inhibition constant of ligands in the binding pocket of caspase-3.

Ligand	Docking Score (ΔG) (Kcal/mol)	Estimated Inhibition Constant (Ki) (µM)	Amino Acid Involved
Isocordoin	−6.13	32.01	Phe256, Trp214, Phe247, Trp 206, Phe250
2,4’-dihydroxychalcone	−5.58	81.57	Phe250, Glu248, Asn208, Trp214, Trp206
NLBA *	−5.52	66.53	Arg207, Tyr204, Asn208, Phe250, Ser209
Daunorubicin	−7.09	6.33	Tyr204, Arg207, Ser205, Phe252
5-FU	−3.68	2000	Asn208, Trp214, Arg207, Phe250

* NLBA: 2-Hydroxy-5-(2-mercaptoethylsulfamoyl)-benzoic acid.

**Table 6:** Physicochemical and lipophilicity of the compounds using Swiss absorption, distribution, metabolism, and excretion (ADME).

Compound	Lipophilicity log P	Heavy Atoms	Aromatic Heavy Atoms	Rot. Bond	H-Bond Acc.	H-Bond Don.	MR ^a^	TPSA ^b^ (A2)	%ABS ^c^
Isocordoin	4.24	23	12	5	3	2	94.01	57.53	89.15
2,4’-dihydroxychalcone	2.75	18	12	3	3	2	70.29	57.53	89.15

^a^ MR: molar refractivity; ^b^ TPSA: topological polar surface area; ^c^ %ABS: percentage of absorption.

**Table 7:** Lipinski drug-likeness and pharmacokinetics parameters of compounds using the Swiss ADME software.

Compound	MW ^a^	Log S ^b^	LV ^c^	Bioavailability Score	GI Absorption ^d^	BBB Permeant ^e^	P-gp Substrate ^f^
Isocordoin	308.37	−5.25	0	0.55	High	Yes	No
2,4’-dihydroxychalcone	240.25	−3.85	0	0.55	High	Yes	No

^a^ MW: molecular weight in g/mol; ^b^ Log S: log of solubility; ^c^ LV: Lipinski violations; ^d^ GI absorption: gastrointestinal absorption; ^e^ BBB permeant: permeation blood-brain barrier; ^f^ P-gp substrate: P-glycoprotein substrate.

## Data Availability

All data are available for scientific community.

## Supplementary Materials

The following supporting information can be downloaded at: https://www.mdpi.com/article/10.3390/ijms26052238/s1.
