# Supplementary material for: In Vitro and In Silico Cytotoxic Activity of Isocordoin from Adesmia balsamica Against Cancer Cells
Source: Int J Mol Sci. 2025 Mar 2;26(5):2238. doi: 10.3390/ijms26052238 (PMC11900625; doi:10.3390/ijms26052238)
Supplement: Supplementary file 1 [file ijms-26-02238-s001.zip › ijms-3477360-supplementary.pdf]

In Vitro and In Silico Cytotoxic Activity of Isocordoin from *Adesmia balsamica* Against Cancer Cells

Valentina Silva [1], Evelyn Muñoz [1], Catalina Ferreira [1], Alessandra Russo [2], Joan Villena [3], Iván Montenegro [4,5], Daniela Birchmeier [6] and Alejandro Madrid [1,5,*]

1. **Spectra S1**. ^{1}H, ^{13}C NMR, MS and IR spectra for isocordoin

Spectra S1. ^{1}H spectra for isocordoin.

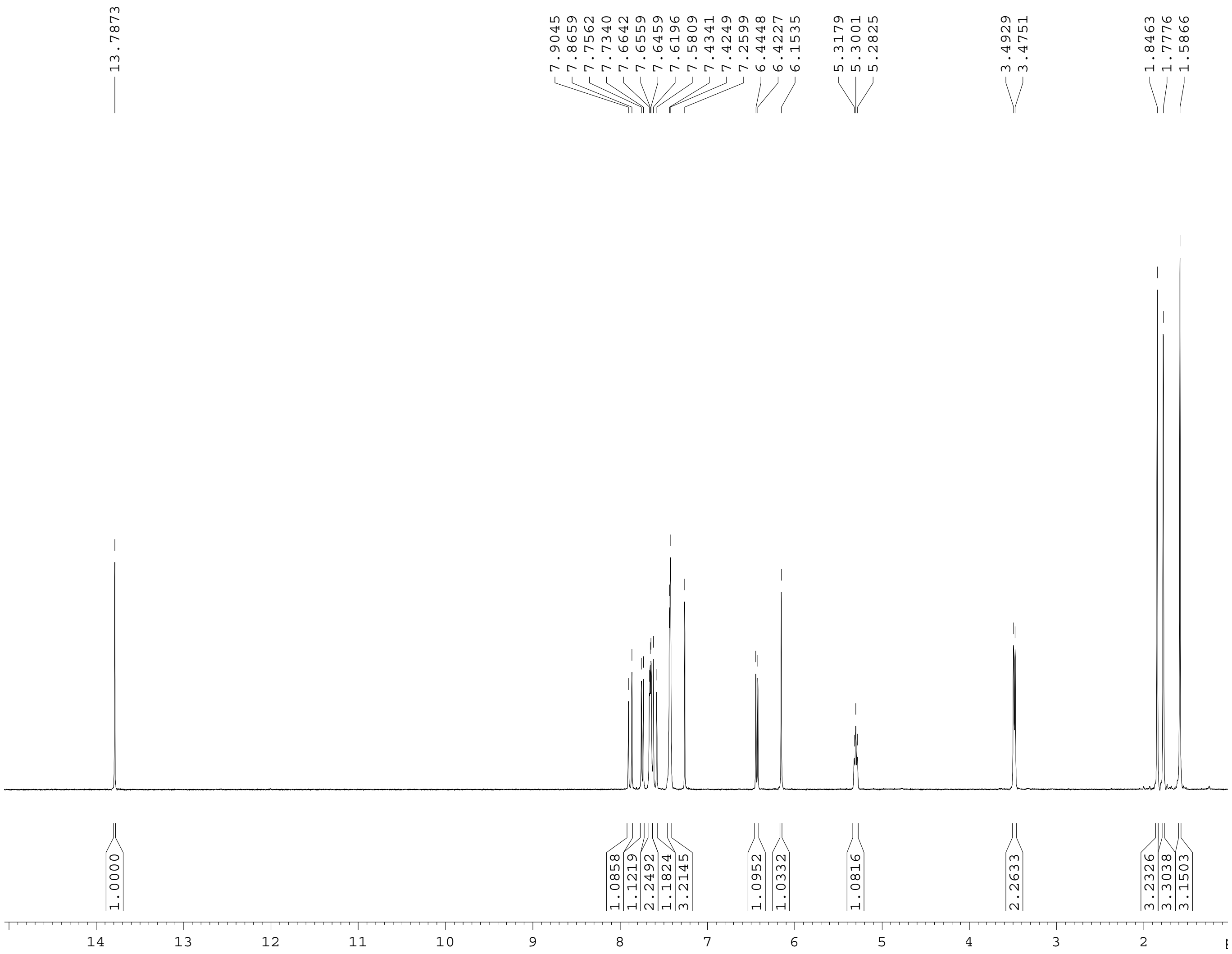

Spectra S2. ^{13}C NMR spectra for isocordoin

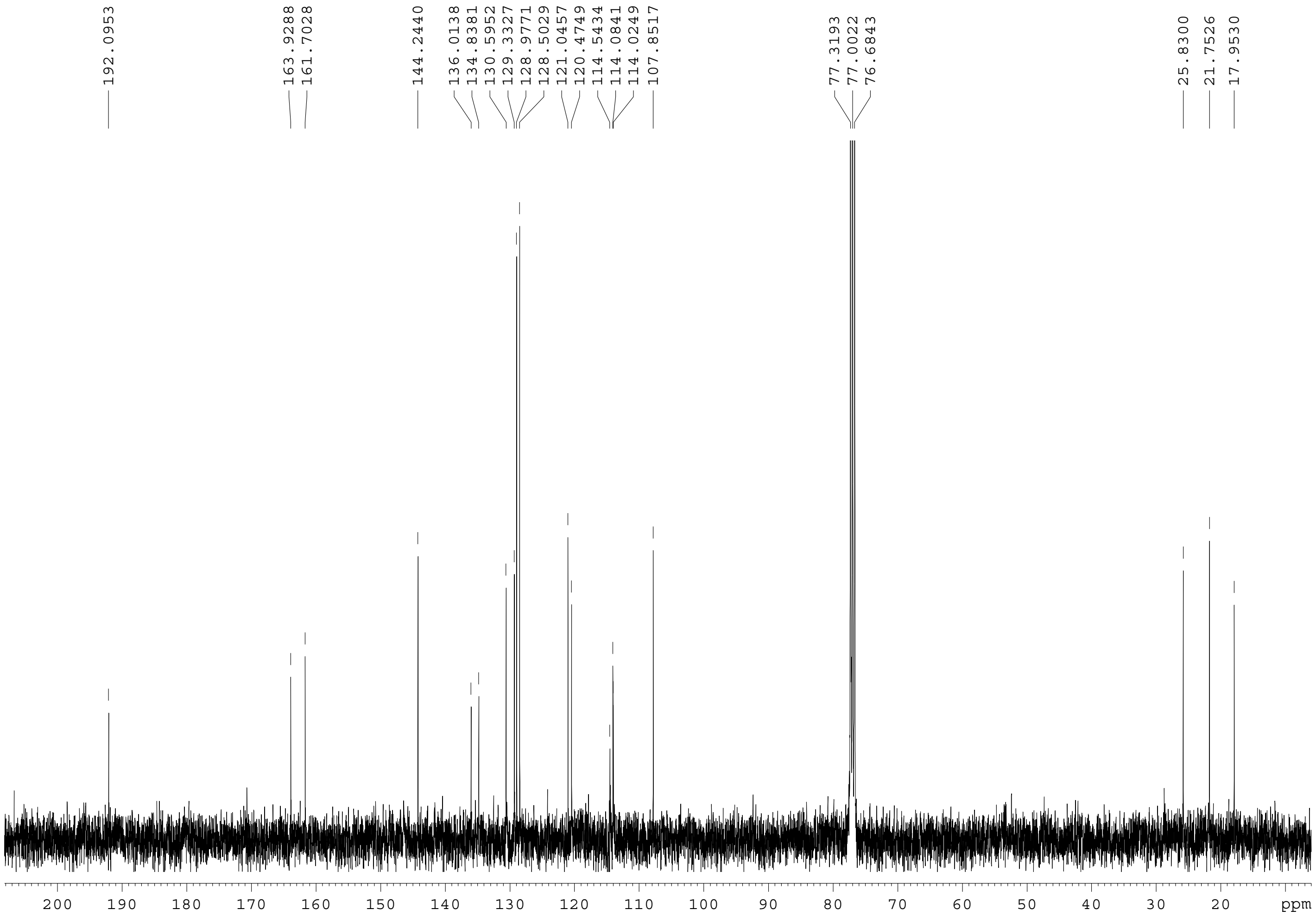

Spectra S3. MS spectra for isocordoin

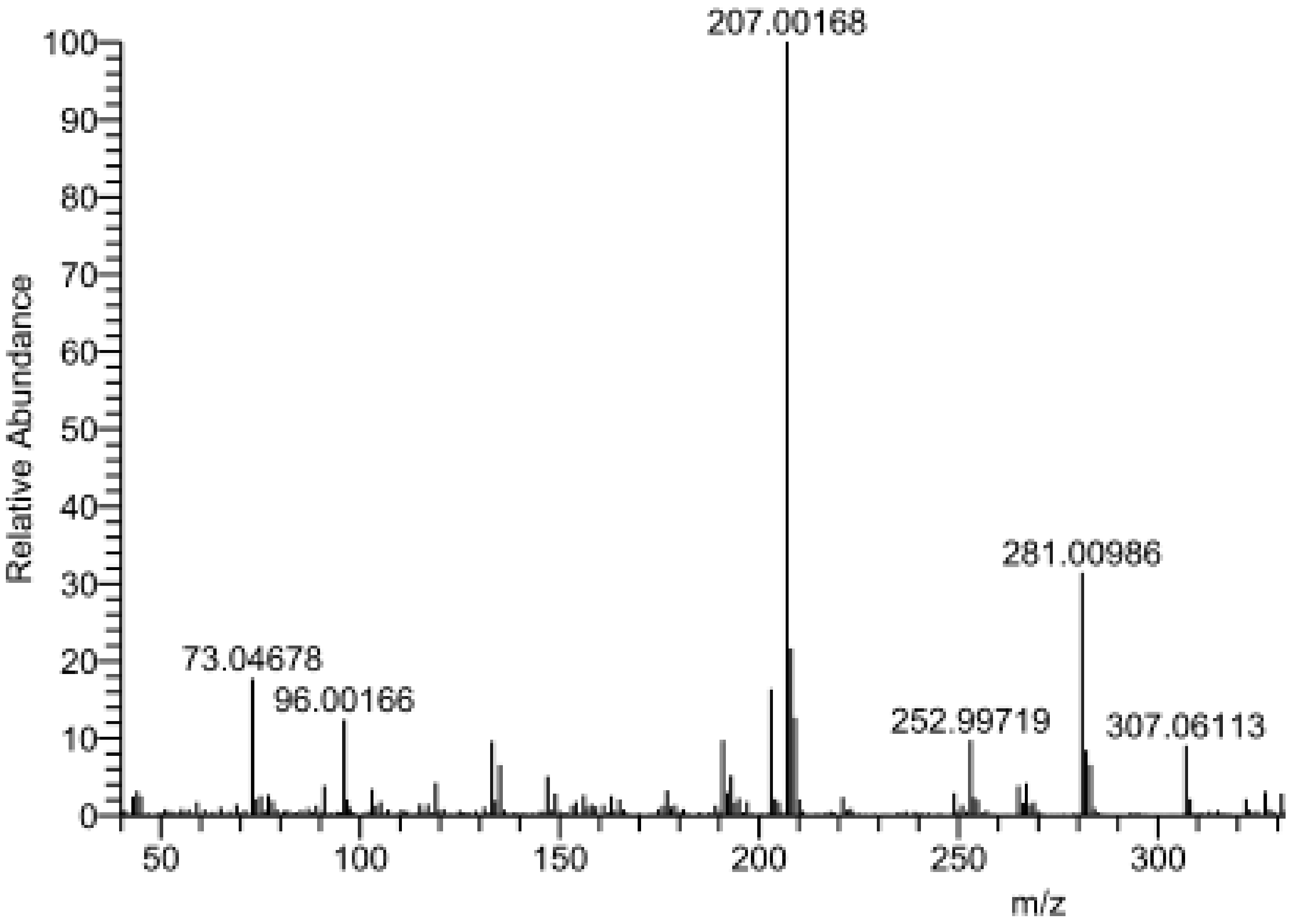

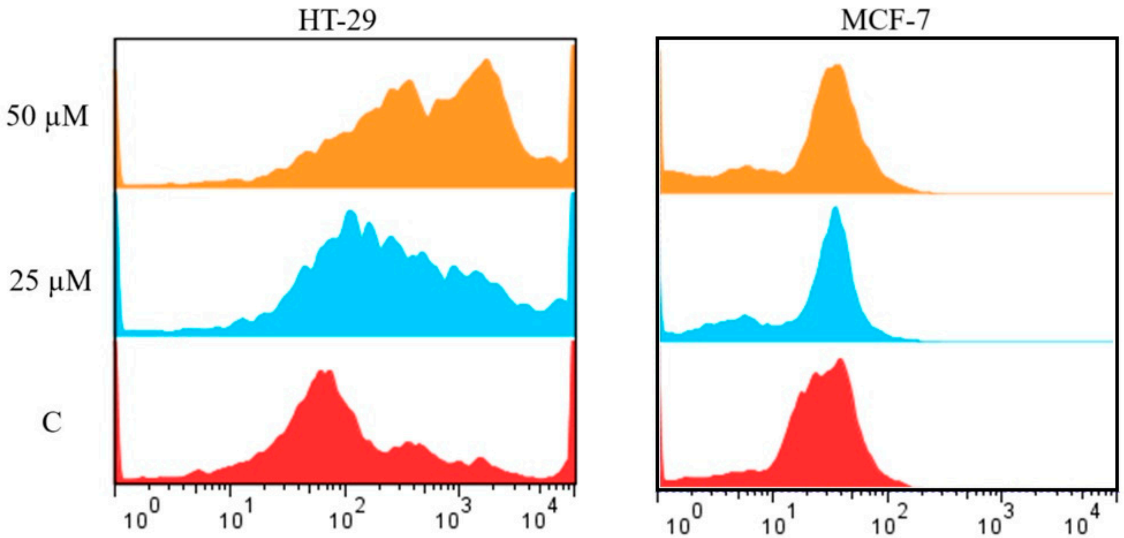

Figure S1 ROS production after treatment with different concentration of isocordoin.

Isocordoin-treated (25 and 50 μM) cells were subjected to flow cytometry analysis. C: control-solvent.

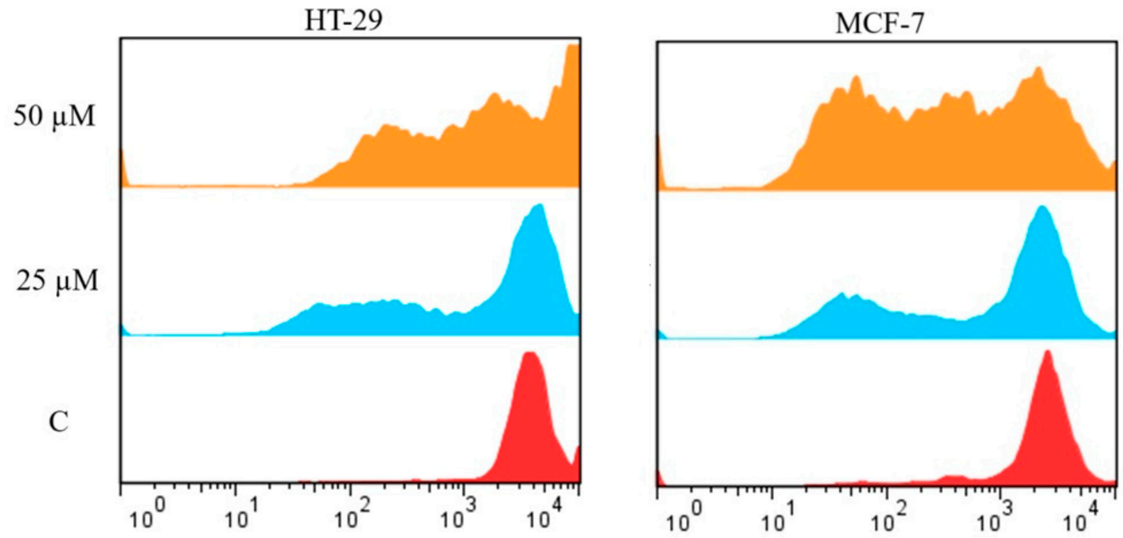

Figure S2 Mitochondrial membrane permeability after treatment with isocordoin (25 and 50 μM). C: ethanol solvent-treated cells.

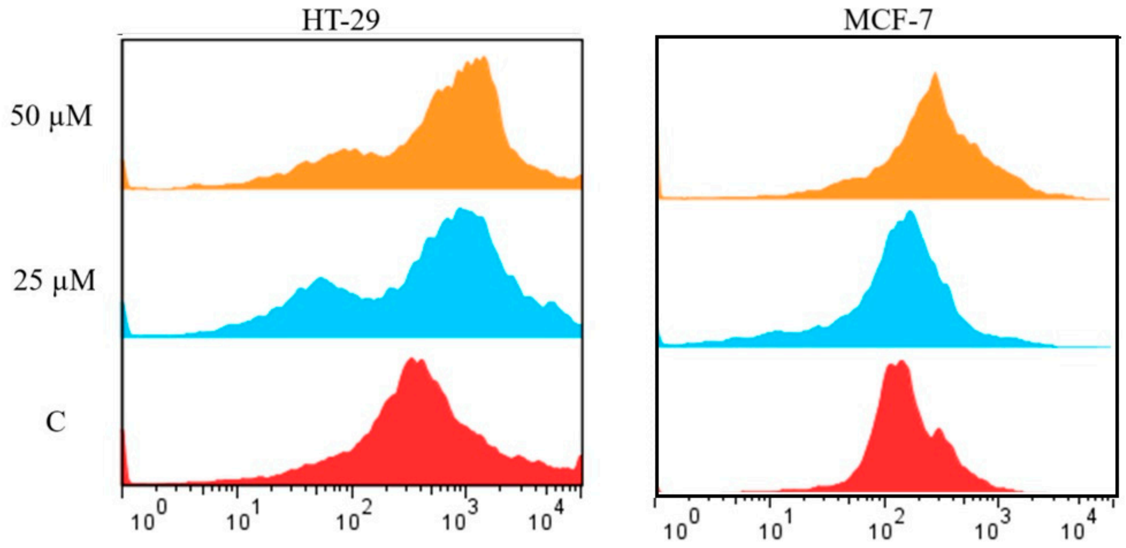

Figure S3 Activation of caspases in HT-29 and MCF-7 cells after treatment with isocordoin (25 and 50 µM). C: ethanol solvent-treated cells.
